# Advances in ciliary proteomics – towards cracking the hidden proteome code of cilia

**DOI:** 10.1242/jcs.264197

**Published:** 2025-10-30

**Authors:** Bernhard Schermer, Ronald Roepman, David U. Mick

**Affiliations:** ^1^ Department II of Internal Medicine, Center for Molecular Medicine Cologne, and Cologne Excellence Cluster on Cellular Stress Responses in Aging-Associated Diseases (CECAD), University of Cologne, Faculty of Medicine and University Hospital Cologne, 50937 Cologne, Germany; ^2^ Department of Human Genetics, Research Institute for Medical Innovation, Radboud University Medical Center, P.O. Box 9101, 6500 HB Nijmegen, The Netherlands; ^3^ Center for Molecular Signaling (PZMS), Department of Medical Biochemistry and Molecular Biology, Saarland University School of Medicine, 66421 Homburg, Germany; ^4^ PharmaScienceHub (PSH), 66123 Saarbrücken, Germany; ^5^ Center for Biophysics (ZBP), Saarland University, 66123 Saarbrücken, Germany

**Keywords:** Cilia, Ciliopathies, Proteomics, Protein networks, Proximity labeling

## Abstract

Understanding the dynamic protein composition of cilia, crucial sensory organelles implicated in ciliopathies, is essential for comprehending cellular function and disease mechanisms. This Review summarizes advancements in methodologies for characterizing cilia proteomes. Traditional biochemical methods for isolating cilia from various model organisms have yielded informative protein inventories of cilia. These approaches worked particularly well for motile cilia but face challenges when investigating sparse primary cilia in various mammalian cells and tissues. The advent of protein–protein interaction mapping approaches employing genetically encoded affinity purification tags followed by mass spectrometry (AP-MS), has elucidated crucial ciliopathy-associated protein modules such as the BBSome, leading to new gene discoveries. More recently, proximity labeling techniques (APEX, BioID and TurboID) have revolutionized the field, enabling high-resolution and dynamic mapping of ciliary proteomes and signaling pathways, and dissecting protein trafficking defects in ciliopathy models. Despite technical variations, these methods offer novel insights into the ciliary and sub-ciliary protein environment. Further advancements in spatial proteomic technologies and mass spectrometry sensitivity might ultimately allow us to delineate dynamic proteomic profiles of specific cilia accurately across different cell types and tissues.

## Teaser

**Summary:** This Review explores proteomic strategies for investigating cilia, tracing the evolution from early discoveries to recent advancements enabled by proximity labeling techniques.

## Introduction

Understanding the protein composition of organelles and their dynamic adaptations is crucial for elucidating how these subcellular compartments and their individual protein complexes contribute to cellular function. Cellular responses to physiological cues bring about dynamic proteomic changes, which also reflect how organelles act as hubs to coordinate and fine-tune complex biological processes. Insights into these processes lay the groundwork for a deeper understanding not only of cellular regulation but also the molecular basis of disease.

### Cilia and the challenge of being small

**Fig. 1.**
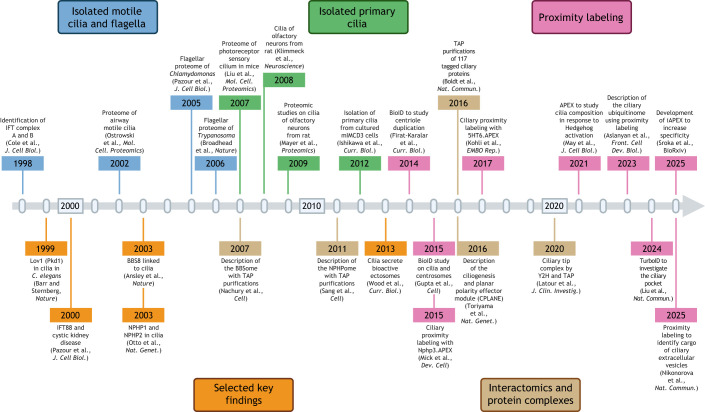
**Cilia research from a proteomics perspective.** Selected key publications significantly shaping our current understanding of cilia. We apologize for not including numerous other relevant studies due to space constraints. Created in BioRender by Schermer, B., 2025. https://BioRender.com/jf2fayw. This figure was sublicensed under CC-BY 4.0 terms.

This complexity is particularly intriguing and especially challenging for cilia, sensory microtubule-based organelles that extend from the cell surface either as solitary structures or in bundles of motile cilia (Pan et al., 2005). Cilia receive and transmit signals and, in their motile forms, generate fluid flow. About 25 years ago, landmark studies first linked cilia to the pathogenesis of cystic kidney diseases – an insight that was later extended to many other genetic syndromes. In 1999, LOV-1 and PKD-2 were shown to localize to cilia in *Caenorhabditis elegans*, the human homologs of which are responsible for autosomal dominant polycystic kidney disease (ADPKD) (Barr and Sternberg, 1999). Around the same time, a mutation in *Tg737*, now termed *Ift88*, a gene essential for ciliary function in *Chlamydomonas reinhardtii*, was found to cause cystic kidney disease in mice (Moyer et al., 1994; Pazour et al., 2000). Soon after, the pediatric cystic kidney diseases nephronophthisis (NPH) and Bardet–Biedl syndrome (BBS) were also causally linked to ciliary dysfunction (see Glossary) (Ansley et al., 2003; Otto et al., 2003). Today, more than 30 disorders with mutations in more than 200 disease genes are classified as primary ‘ciliopathies’ (Turan et al., 2023). With the exception of ADPKD, these are rare disorders and syndromes with a broad genotypic and phenotypic overlap. They currently lack any registered curative therapies, underscoring the urgency of gaining a deeper understanding of their underlying pathological mechanisms. In recent decades, research has significantly focused on characterizing ciliary protein complexes and elucidating the protein composition of cilia (Fig. 1). These approaches are based on the rationale that comprehensively decoding the content and complexity of cilia will lead to a deeper understanding of their function and pathophysiological roles.

Glossary **Cilia structural terms**

**Axoneme:** the microtubule-based core of the cilium.

**Basal body:** modified mother centriole that forms the base of the cilium.

**BBSome:** multiprotein complex composed of eight Bardet–Biedl syndrome (BBS) proteins that mediates the trafficking of membrane proteins out of the cilium.

**Central apparatus:** a structure within motile cilia and flagella composed of two central microtubules surrounded by protein complexes, playing a key role in coordinated ciliary beating.

**CPLANE:** the ciliogenesis and planar polarity effector complex, a multiprotein assembly at the ciliary base, essential for proper ciliogenesis and planar cell polarity.

**IFT complexes:** intraflagellar transport (IFT) complexes are multi-protein complexes that mediate the bidirectional movement of cargo along the axonemal microtubules, essential for cilium assembly, maintenance, disassembly and signaling.

**Transition zone:** specialized region between the basal body and the ciliary axoneme that regulates entry and exit of proteins.

**Proteomics terms**

**Affinity purification–mass spectrometry (AP-MS):** approach in which proteins of interest are purified together with their interaction partners using affinity tags followed by mass spectrometric analysis.

**Immunoprecipitation–mass spectrometry (IP-MS):** approach in which proteins of interest are purified together with their interaction partners using specific antibodies, followed by mass spectrometric analysis.

**Tandem-affinity purification (TAP):** protein purification technique in which a target protein is fused to two affinity tags, allowing sequential purification steps.

**Localization and affinity purification (LAP):** special version of the TAP technique, in which one tag is a fluorescent protein that can be detected by fluorescence, which allows two-step purification and localization studies.

**Proximity-dependent biotin identification (BioID):** a method based on a promiscuous biotin ligase (BirA*) fused to a protein of interest to covalently label neighboring proteins with biotin for subsequent affinity purification and mass spectrometric analysis.

**TurboID:** an engineered biotin ligase with enhanced activity compared to BioID. A shorter truncated version is referred to as miniTurboID.

**Engineered ascorbate peroxidase (APEX):** a proximity labeling enzyme that uses biotin-phenol and hydrogen peroxide to biotinylate nearby proteins for subsequent purification and mass spectrometric analysis.

**Ciliopathies**

**Nephronophthisis (NPH):** an autosomal-recessive cystic kidney disease characterized by progressive tubulointerstitial fibrosis, corticomedullary cyst formation, and eventual kidney failure, often accompanied by extrarenal manifestations. NPH is caused by mutations in >20 NPHP genes.

**Joubert syndrome (JBTS):** a rare neuronal ciliopathy defined by the “molar tooth sign” on brain MRI, reflecting cerebellar vermis hypoplasia. JBTS presents with variable neurological, ocular, renal, hepatic and skeletal manifestations and is genetically heterogeneous, caused by mutations in more than >35 ciliary genes, many of which overlap with NPH and BBS. JBTS is also classified within the group of NPH-related ciliopathies (NPH-RC).

**Meckel–Gruber syndrome (MKS):** a severe, typically lethal ciliopathy characterized by occipital encephalocele, cystic kidneys, hepatic fibrosis and polydactyly. MKS is caused by mutations in multiple ciliary genes, many of which overlap with those implicated in Joubert syndrome and related disorders. MKS is also classified within the group of nephronophthisis-related ciliopathies (NPH-RC).

**Bardet–Biedl syndrome (BBS):** a pleiotropic ciliopathy characterized by retinal degeneration, obesity, polydactyly, cognitive impairment, hypogonadism and kidney dysfunction. BBS is caused by mutations in BBS genes encoding components of the BBSome, a chaperone complex and additional ciliary proteins. It is also classified among the nephronophthisis-related ciliopathies (NPH-RC).

**Autosomal dominant polycystic kidney disease (ADPKD):** the most common inherited kidney disorder, caused by mutations in PKD1 or PKD2, leading to progressive cyst formation and kidney enlargement.

Cilia are composed of a microtubule-based skeleton, termed the axoneme (see Glossary), which is typically arranged in a ring of nine doublet microtubules that – in the case of most motile cilia – surrounds two central single microtubules (Walton et al., 2023). However, this canonical structure can show pronounced deviations with increasing distance from the ciliary base (Kiesel et al., 2020). The basal body (see Glossary) is a modified mother centriole, which anchors and provides the structural template for the primary cilium (Kim and Dynlacht, 2013). Situated just above the basal body is the transition zone (see Glossary), which acts as a selective barrier and regulates protein entry and exit, as both membrane and cytoplasmic proteins cannot freely access the cilium (reviewed in Moran et al., 2024; Nachury and Mick, 2019).

A major challenge in cilia research, particularly in analyzing the ciliary protein composition, is the extremely small volume of cilia compared to the rest of the cell. This limitation is further compounded in the case of primary cilia, where only one cilium is typically formed per cell. A single cilium has a diameter of ∼200–250 nm and a length of 1–10 µm, corresponding to a volume in the order of tenths of a femtoliter (Polino et al., 2023). Despite established protocols for mammalian primary cilia isolation (Huang et al., 2009; Ishikawa and Marshall, 2013; Mitchell, 2013; Mitchell et al., 2009), obtaining sufficient material at adequate purity for proteomic analyses remains technically demanding.

### Proteomic approaches for studying cilia

**Fig. 2.**
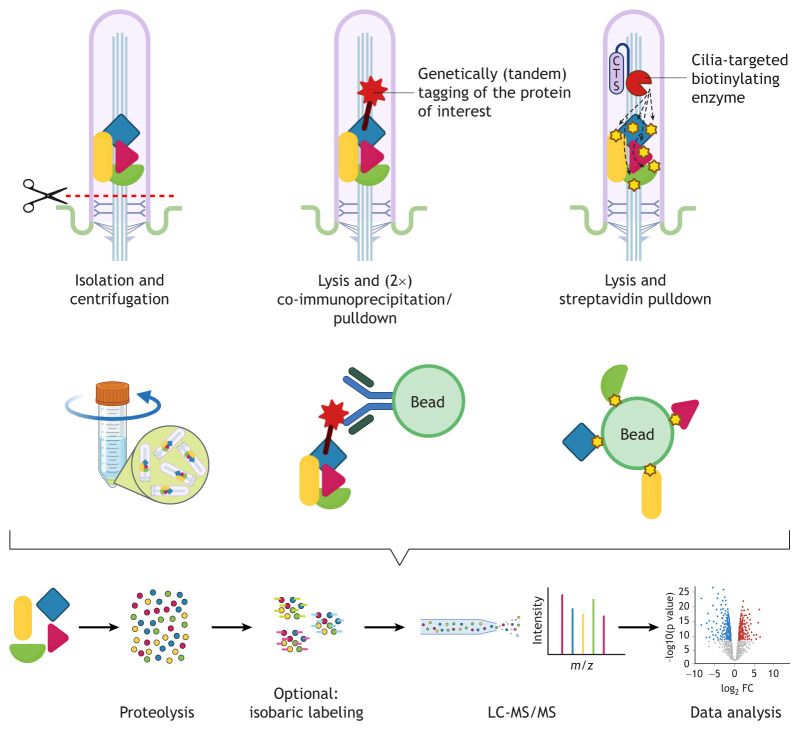
**Three basic experimental principles for proteomic studies of cilia.** Left, isolation of intact cilia by chemical or physical means followed by purification and proteomic profiling. Colored shapes represent ciliary proteins of interest. Middle, approaches aimed at investigating the interactome of ciliary proteins, commonly employing genetically tagged proteins combined with single-step or tandem affinity purification, especially when suitable antibodies are not available. Right, proximity labeling techniques based on targeting biotinylating enzymes specifically to cilia, followed by metabolic labeling and streptavidin-based pulldown. Bottom, all methodologies conclude with proteomic analysis. Isolated proteins of interest undergo proteolysis, and proteomic analysis is typically performed using isobaric labeling [e.g. tandem mass tag (TMT)] or label-free quantification (LFQ). Samples are analyzed using liquid chromatography tandem mass spectrometry (LC-MS/MS) to generate data for analysis. Created in BioRender by Schermer, B., 2025. https://BioRender.com/cmiv19x. This figure was sublicensed under CC-BY 4.0 terms.

Approaches in ciliary proteomics began with the straightforward strategy of biochemically isolating flagella and cilia and analyzing their protein content by mass spectrometry (Fig. 2; Box 1). Subsequent efforts shifted toward interaction studies of known ciliary proteins using pulldown and immunoprecipitation experiments (IP-MS, see Glossary), with tandem affinity purification playing a central role. More recently, ciliary proximity labeling – introducing biotinylating enzymes into the cilium – has emerged as a powerful tool for probing ciliary proteomes. It is noteworthy, although not central to the focus of this Review, that there are numerous studies that have made crucial contributions by employing comparative genomics (Avidor-Reiss et al., 2004; Li et al., 2004), siRNA (Failler et al., 2021; Kim et al., 2016; Lai et al., 2011; Roosing et al., 2015; Szymanska et al., 2015) or CRISPR-based screens (Breslow et al., 2018; Pusapati et al., 2018) to identify genes essential for ciliogenesis, ciliary disassembly, cilia-mediated signal transduction or other aspects of cilia biology. The abovementioned proteomics analyses and comparative genomics studies provided the first inventories of cilia, revealing up to 1200 distinct proteins comprising cilia in different biological contexts (Avidor-Reiss et al., 2004; Gherman et al., 2006; Li et al., 2004). These studies further highlight the primary cilium as a sensory organelle and provide a basic understanding of how its dysfunction can cause disease (Singla and Reiter, 2006).

Box 1. Mass spectrometry advancements aiding ciliary proteomics There are many valid approaches to perform mass spectrometry on cilia. The choice of instrument depends on the samples to be analyzed and the downstream analysis (Jiang et al., 2024). Co-immunoprecipitation (co-IP) experiments can provide very clean, low-complexity samples that can be analyzed on any state-of-the-art instrument. Proximity labeling approaches, however, generate more complex samples, in which the ciliary proteins are often the needles in the haystack to be identified and, ideally, quantified. To identify bona fide cilia proteins from unwanted background, control samples lacking cilia proteins are of utmost importance. Valid controls include not performing proximity labeling reactions, mis-localizing the proximity labeling enzymes or ablating cilia completely (Aslanyan et al., 2023; Kohli et al., 2017; May et al., 2021). High sample complexity makes quantifying low-abundance cilia proteins very challenging.

Two conceptually different approaches for quantification have been utilized for primary cilia: (1) label-free quantitation methods, which normalize systematic biases between samples to determine the relative amounts of identified peptides from their ion intensities (Ammar et al., 2023) and (2) label-based quantification, where peptides from individual samples are modified by defined masses, such that different samples can be analyzed and quantified in parallel, known as multiplexing (Li et al., 2021).

Label-free quantification methods avoid issues faced with multiplexing, such as lowered sensitivity due to increased sample complexity. Label-free methods are very precise for a broad range of protein abundances. However, for proteins with very low abundance near the detection limit, minimal signals might be detected in one but not all samples, which further complicates quantification. Label-based quantification, especially by so-called tandem mass tags, almost completely abolishes this problem, as identified peptides will be further fragmented to determine relative protein abundances across all multiplexed samples, such that quantification of almost every identified peptide is possible. Recent developments in mass spectrometry have brought us faster and more sensitive instruments with huge impacts on sample throughput. Additionally, advanced data processing software has boosted sensitivity for label-free proteomics through switching from data-dependent acquisition to data-independent acquisition (Guzman et al., 2024). Although these approaches are quickly becoming the standard for whole-cell proteomics, it will be exciting to see their performance in comparative cilia proteomics.

Here, we review recent advances in ciliary proteomics and place them in a historical context. Our focus is on primary cilia because our knowledge on the proteomes of motile cilia is already more comprehensive. This is largely due to the relative ease of isolating motile cilia and flagella in greater quantities from model organisms such as *Chlamydomonas* and mammalian tissues, making them more accessible to mass spectrometry-based analyses. By contrast, primary cilia pose significant experimental challenges due to their size and numbers, necessitating the development and implementation of advanced methodology. We first highlight insights gained from mass spectrometry analyses of isolated flagella and cilia. We then discuss protein–protein interaction networks revealed by affinity purification–mass spectrometry (AP-MS), using ciliary and ciliopathy-associated proteins as baits. Finally, we examine how proximity labeling approaches have been applied to study ciliary function, outlining the strategies used to date and concluding with perspectives on future efforts to further elucidate the molecular composition of cilia.

### Proteomic insights from mass spectrometry on isolated cilia

The most direct approach to analyzing the protein composition of cilia is through biochemical isolation and purification, followed by mass spectrometry-based proteomic analysis (Fig. 2). This strategy has proven particularly successful for motile cilia and flagella, owing to their relatively large quantities. Studies in *Chlamydomonas reinhardtii*, which uses its two flagella both for locomotion and reproduction, include a comprehensive flagellar proteome (Pazour et al., 2005), a phosphoproteome (Boesger et al., 2009), and detailed proteomic characterizations of the transition zone (Diener et al., 2015), the central apparatus (Zhao et al., 2019) and, more specifically, the central microtubule pair of the axoneme (Dai et al., 2020). More recently, mass spectrometry has also been applied to investigate the roles of transition zone proteins in regulating ciliary composition (Wang et al., 2022). Equally notable are studies on the flagella of *Trypanosoma* (Broadhead et al., 2006; Subota et al., 2014) and on motile cilia in *Xenopus* embryos (Sim et al., 2020). In mammals, early proteomic efforts focused on human airway epithelial cells (Blackburn et al., 2017; Ostrowski et al., 2002). More recently, similar analyses have been extended to motile cilia in brain ventricles, the oviduct and sperm, including human samples (Leung et al., 2025).

By contrast, proteomic studies of primary cilia remain limited, largely due to technical challenges associated with their small size and low abundance. The earliest proteomic investigation of a primary cilium was on the specialized sensory cilium of retinal photoreceptor cells – also referred to as the photoreceptor outer segment – in the mouse retina (Liu et al., 2007). In this study, isolated photoreceptors were analyzed, including a mouse model lacking ciliary rootlets, cytoskeletal filamentous structures that emanate from the basal body into the cytoplasm and typically co-purify as contaminants of photoreceptor outer segment preparations. The datasets revealed known cilia-associated proteins as well as several ciliopathy-related proteins including several BBS or NPHP proteins that had not previously been reported in photoreceptors. As a key reference for cilia-associated proteins at that time, the study employed the ‘ciliary proteome database’, a comprehensive database from 2006 largely derived from transcriptomic data of ciliated cells and proteomic data from flagella across ten prior studies (Gherman et al., 2006). Subsequent studies analyzed ciliary membranes isolated from olfactory sensory neurons in rats and mice, identifying several hundred proteins that can be grouped, among others, into signal transduction, Ca^2+^ signaling and cytoprotective pathways (Klimmeck et al., 2008; Mayer et al., 2008, 2009; Stephan et al., 2009). Methodological advances in cilia isolation and membrane protein enrichment later enabled the identification of over 4400 proteins from mouse olfactory epithelia, including a substantial number of olfactory receptors (178 proteins) within the olfactory ciliary membrane proteome (Kuhlmann et al., 2014). Another study examined the ciliary proteome of choroid plexus cells (Narita et al., 2012), which harbor multiple so-called 9+0 cilia that lack the central microtubule pair and exhibit features of both primary and motile cilia (Ho et al., 2023). In the first study to investigate canonical primary cilia from cultured cells, primary cilia were isolated from the murine inner medullary collecting duct cell line mIMCD3 (Ishikawa and Marshall, 2013), a well-established model for kidney epithelial cells, identifying 195 candidate primary cilium proteins (Ishikawa et al., 2012). In addition to strategies using mass spectrometric approaches, a recent study employed spatial proteomics based on antibody staining to identify proteins with ciliary localization. This effort mapped 650 ciliary proteins, including 71 candidates previously not linked to cilia, and revealed notable differences in ciliary protein composition across different cell lines (Hansen et al., 2025).

## Protein–protein interaction mapping approaches to investigate ciliopathy-associated protein networks

### The importance of understanding cilia protein–protein interactions

Affinity purification-based mass spectrometry techniques have enabled the study of protein–protein interactions (PPIs), providing valuable insight into disease. In order to understand the structure and function of a cell and its dysfunction upon genetic mutation, it is crucial to obtain a detailed, comprehensive view of the network of interactions between its many constituents (Vidal et al., 2011). PPIs and the composition of protein complexes are inherently dynamic in nature and adjusting individual components in response to environmental signals provides flexibility in function. This allows cells to adapt to changing conditions and generate the required robustness that is critical for life (Barabasi and Oltvai, 2004). By contrast, even a subtle disturbance of a crucial PPI (e.g. from a single missense variant) can have significant systemic consequences by affecting individual protein modules and interconnected cellular networks, which can cause disease (Barabasi et al., 2011).

The advent of rapid sequencing technologies has significantly increased the capacity to identify pathogenic genetic variants in individuals with ciliopathies and scrutinize the molecular mechanisms of disease (Oud et al., 2017). This boost in identification of novel ciliopathy genes has revealed ciliary proteins that are localized to or affect the biogenesis or function of cilia (Hildebrandt et al., 2011; Mill et al., 2023; Quinlan et al., 2008). These include key ciliary proteins, such as those involved in intraflagellar transport (IFT, see Glossary) that have been well-studied in *C. reinhardtii* and *C. elegans* (Barr et al., 2001; Cole et al., 1998; Pazour et al., 2000). Yet, the ciliary roles of many other ciliopathy genes remain elusive. As the initial defect in monogenic ciliopathies is a single disrupted – or even absent – protein, characterizing its interaction network is intuitively crucial to explain pathogenicity as it follows the ‘guilt-by-association’ paradigm (Wang and Marcotte, 2010). This concept is based on the idea that the function of a protein often resembles that of the proteins it interacts with or shares other features with, such as co-expression and colocalization (Hartwell et al., 1999). This theoretical concept has been experimentally validated in several seminal studies across eukaryotes of increasing complexity, from yeast and *C. elegans* to humans (Bork et al., 2004; Gavin et al., 2006).

An extensive and ever-growing toolbox of molecular techniques is available to identify, map and study PPIs and protein complexes (Snider et al., 2015). Most methods are based on ‘bait–prey’ principles. These enable the identification of binary interactors, such as classic yeast two-hybrid (Y2H) approaches, or the isolation of protein complexes by affinity purification of a specific ‘bait’ protein, followed by mass spectrometric identification of the isolated associated ‘preys’ (AP-MS or affinity proteomics; see Fig. 2 and Glossary). Both approaches have been utilized in large-scale screens to map the human interactome (Huttlin et al., 2015; Rual et al., 2005). To understand the function of ciliopathy-associated proteins and to identify novel ciliopathy candidate genes, several studies have dissected PPI networks using ciliary and ciliopathy-associated proteins as ‘baits’. These studies generated lists of protein modules that informed us on the molecular functions of the primary cilium as a signaling organelle. Prior to 2005, Y2H screens of tissue-specific cDNA libraries were laborious-but-efficient tools to identify binary interactors of ciliopathy-associated proteins derived from genetic studies. This elucidated, for example, the ciliary protein module linked to the retinitis pigmentosa GTPase regulator (RPGR; involved in X-linked retinitis pigmentosa), which contains several overlapping retinal, renal and neuronal ciliopathy-associated proteins including RPGRIP1 (Roepman et al., 2000), RPGRIP1L (Arts et al., 2007), NPHP4 (Roepman et al., 2005) and SPATA7 (Eblimit et al., 2015). The same approach also linked nephrocystin-5, involved in the retinal–renal ciliopathy Senior–Løken syndrome, to RPGR and calmodulin (Otto et al., 2005).

### Tandem affinity purification

Although informative, the stepwise Y2H screening approaches are relatively slow and require several validation steps, as the interactors are not physically purified from cells or tissues. Mass spectrometry-based affinity proteomics approaches have improved the mapping of ciliopathy-associated PPI networks. Owing to the small size of primary cilia, it is important to increase the specificity of the isolated protein complexes when developing techniques by reducing unwanted background (Gingras et al., 2005). The development of a method that fuses an epitope tag to the bait protein has allowed for two consecutive affinity purifications (tandem affinity purification, TAP; Glossary) (Rigaut et al., 1999), enabling systematic PPI analysis under near-physiological conditions in various organisms (Puig et al., 2001). Several flavors of this procedure have been developed and used to identify and dissect ciliary protein complexes.

### Localization and affinity purification

A version of TAP with an epitope tag that includes a GFP domain, a protease cleavage site and an S-peptide tag, has enabled efficient monitoring of the localization and affinity purification (LAP; see Glossary) of the target protein and its associated protein complex. This approach was utilized in the identification of an octameric protein module disrupted in many individuals with Bardet–Biedl syndrome, the BBSome (see Glossary) (Loktev et al., 2008; Nachury et al., 2007), using one of the known BBS-associated proteins, BBS4, as a bait. The same method was subsequently used to unravel the functional modules associated with nine ciliopathy-associated bait proteins, NPHP1–NPHP6, NPHP8, AHI1 and MKS1, identified in the ciliopathies nephronophthisis (NPH), Joubert syndrome (JBTS) and Meckel–Gruber syndrome (MKS) (see Glossary). These proteins were found to be stably expressed in three different ciliated cell lines, hTERT-RPE1, NIH-3T3 and mIMCD3, in a total of 15 affinity purification protein mass spectrometry experiments (Sang et al., 2011). This yielded an integrated NPHP-JBTS-MKS protein network, with functionally linked but spatially separated protein modules identified at the basal body, the transition zone and the so-called inversin compartment. Following the ‘guilt-by-association’ paradigm, 38 interactors from this network were screened for mutations in a ciliopathy patient cohort. Two new ciliopathy disease genes were identified: *ATX10*, mutated in individuals with NPH, and *TCTN2*, associated with JBTS (Sang et al., 2011).

Employing the LAP approach with additional baits subsequently detailed the ciliary protein complexes that form the transition zone barrier, most members of which are associated with ciliopathies with their disruption predominantly causing defects in ciliogenesis and Sonic hedgehog signaling (Chih et al., 2011; Garcia-Gonzalo et al., 2011). LAP-based IP-MS of the cilium-associated trafficking protein TULP3 has linked it to the intraflagellar transport complex IFT-A, revealing how G-protein-coupled receptors (GPCRs) are targeted into cilia (Mukhopadhyay et al., 2010). LAP-based IP-MS also enabled identification of a ‘ciliogenesis and planar polarity effector’ (CPLANE, see Glossary) module at the ciliary base, which includes planar cell polarity-associated proteins Inturned, Fuzzy and Wdpcp, the protein JBTS17 (also known as CPLANE1) and the GTPase RSG1 (also known as CPLANE2) (Toriyama et al., 2016). The genes encoding the five CPLANE proteins are mutated in multiple overlapping ciliopathies, including short-rib polydactyly syndrome (SRPS), BBS, JBTS, MKS and Oral-facial-digital syndrome (OFD) (Toriyama et al., 2016; Vazquez et al., 2025). The crescent-shaped structure of the CPLANE complex was recently elucidated by cryo-EM and seems to facilitate interactions with small GTPases (Rab23 and Rsg1) and phosphatidylinositol monophosphates (Langousis et al., 2022), providing insights into CPLANE dysfunction in ciliopathies.

### Strep–Flag tandem affinity purification

To reduce the complexity of the TAP procedure for high-throughput procedures, we have fused bait proteins of interest with either an N- or C-terminal TAP sequence that includes a double Strep II epitope (binding to Strep-Tactin) and a Flag epitope (binding to anti-Flag) (Boldt et al., 2009; Gloeckner et al., 2007). The small size (4.6 kDa) of the synthetic and hydrophilic moieties of the Strep–Flag (SF) tag greatly reduces nonspecific protein binding as well as steric hindrance. By using competitive elution instead of a proteolytic cleavage step, this tag allows the undisturbed isolation of native protein complexes in relatively short times. This efficient SF-TAP method has enabled us to perform a systems biology approach to understand cilium function by providing insights into the protein landscape of cilia through large-scale SF-TAP-MS of 217 bait proteins with ciliary and/or ciliopathy associations (Boldt et al., 2016). This yielded an interactome that encompassed 1319 proteins and 4905 interactions that could be assembled into 52 complexes, containing ∼60% of all ciliopathy-associated proteins known at the time. Moreover, our study pinpointed ciliary associations to many other protein networks, including those involved in 3-M syndrome, a disease not previously identified as having ciliary involvement (Boldt et al., 2016). By adapting a quantitative measure of PPIs, termed the socioaffinity index, new experimental data could be mapped to the interactome, which provided relational insights to ciliary protein function and their dysfunction in ciliopathies (Aloy et al., 2004; Boldt et al., 2016; Gavin et al., 2006). As such, a combination of Y2H screening and SF-TAP-MS discovered a complex of five Joubert syndrome-associated proteins (CEP104, CSPP1, TOGARAM1, ARMC9 and CCDC66) at the primary cilia tip (Latour et al., 2020). Recently, cryo-EM and *in vitro* microtubule reconstitution assays has revealed that this complex forms microtubule plus-end-specific cork-like structures that reduce protofilament flaring (Saunders et al., 2025). Flaring describes the outward splaying of microtubule protofilaments at the ends of microtubules (McIntosh et al., 2008), a structural feature intricately connected to the slow growth and stability of centriolar microtubules (Iyer et al., 2025). Reduction of protofilament flaring thus imparts slow and processive microtubule growth required for accurate ciliary length regulation (Saunders et al., 2025).

The SF-TAP approach has also been instrumental for deciphering the complex interplay of proteins present in the connecting cilia of the rod and cone photoreceptor cells in the retina. Connecting cilia are mainly the extended transition zones of the sensory cilia of photoreceptors (Liu et al., 2007). These are highly specialized primary cilia of our body as they are optimized for phototransduction and vision. One of the key connecting cilia proteins is lebercilin, encoded by the gene *LCA5*, whose function is disrupted in a condition causing congenital blindness (Leber congenital amaurosis type 5, LCA5). Our SF-TAP studies revealed that lebercilin recruits several protein modules, including the IFT-B complex (den Hollander et al., 2007). The association with the IFT-B module was lost due to *LCA5*-associated gene mutations that disrupted lebercilin function, which was determined by stable isotope labeling of amino acids in cell culture (SILAC) that allowed quantitative AP-MS (Boldt et al., 2011). This provided two hypotheses about the lebercilin protein complex interplay at the specialized photoreceptor axoneme stalk: (1) lebercilin provides a scaffold for IFT proteins, (2) IFT traffics lebercilin across the ciliary axoneme, or both. Recently, we visualized this interplay in the mouse retina (Faber et al., 2023). Inducing Flag–lebercilin expression in the mouse retina by intravitreal injection of an adeno-associated virus (AAV) gene therapy vector enabled both retinal proteomics (Flag-based AP-MS) and ultrastructure expansion microscopy-based high-resolution imaging of mouse retinas (Faber et al., 2023). Our study validated the hypothesis that lebercilin provides an IFT protein scaffold at the tip of the axoneme stalk of the photoreceptor sensory cilium, which is crucial for its development. Moreover, the loss of this function due to *LCA5* mutations could be rescued by *LCA5* gene augmentation (Faber et al., 2023).

Further improvements of the specificity and relevance of the described AP-MS approaches are focused on dissecting ciliary proteomes in specific cells and tissues. These include placing the transgene under control of a cell type-specific promotor upon expression in zebrafish using a Tol2 transposon system, as performed for whirlin in photoreceptor cells (Schellens et al., 2022). Alternatively, endogenous tagging approaches have been deployed in cells (Beyer et al., 2018) and in mice (Beyer et al., 2024). Here, CRISPR/Cas9-based genome editing is used to include the sequences encoding the epitope tags with the open reading frame of the endogenous target gene. This prevents artifacts due to the overexpression of a transgene (Gibson et al., 2013) and thus bears great potential for future mechanistic and cell type-specific studies.

Taken together, AP-MS approaches played major roles in the original identification as well as the functional characterization of ciliary protein networks. This has required data from manifold experiments and different laboratories to paint a wholistic picture of the cilium and the cilia proteome.

## Using proximity labeling to understand cilia function

Since 2012, proximity labeling techniques have revolutionized subcellular proteomics and the study of PPIs (Rhee et al., 2013; Roux et al., 2012). Specific enzymes are directed to subcellular locations, where they attach molecular handles on proteins, which are then used to isolate and identify them by mass spectrometry. Among these techniques, ascorbate peroxidase (APEX) and biotin identification (BioID) (see Glossary) and their subsequent improvements, APEX2 (Lam et al., 2015), TurboID and miniTurbo (Branon et al., 2018), have emerged as the most common tools for mapping proteomes and interactomes with high spatial and temporal resolution. Although the underlying (bio)chemistry – such as their substrates and enzymatic activities – differ between APEX- and BioID-based strategies, both technologies have been instrumental in determining the proteomes of primary cilia and the centrosome–cilium complex. Here, we discuss the insights these techniques have provided into different aspects of cilia biology.

### Cilia structure

The first studies using proximity labeling mapped the centrosomes as well as primary cilia from commonly used cell lines (U2-OS, HEK, mIMCD3) (Firat-Karalar et al., 2014; Kohli et al., 2017; Mick et al., 2015). Focusing on primary cilia, two studies have applied APEX-based proximity labeling and proteomics in mIMCD3 kidney epithelial cells, and identified established cilia proteins while also revealing interesting differences, specifically pertaining to actin regulators and proteins involved in signal transduction (Kohli et al., 2017; Mick et al., 2015). Given that both studies used the same cell line model, the observed variations were likely due to technical differences, including in MS methods (Box 1), but also in how the APEX enzymes were targeted to primary cilia (see Fig. 2). In addition to identifying known structural components, our first study (Mick et al., 2015) performed proteomic profiling in a BBS model cell line, in which *Ift27* (also known as *Bbs19*) was disrupted, and recapitulated the accumulation of the BBSome as well as GPCRs in the *Ift27* mutant cilia (Eguether et al., 2014; Liew et al., 2014). In contrast, the model employed by Kohli et al. provided insights into how actin regulators are involved in cilium length regulation and ciliogenesis (Kohli et al., 2017). Another study used BioID to determine proximity maps of 56 proteins at the centrosome–cilium complex and revealed extensive networks and considerable interplay between many ciliary and centrosomal components (Gupta et al., 2015). That study confirmed the central role of centriolar satellites in the assembly of primary cilia, whereas proximity proteomics revealed further centrosome-independent functions (Gheiratmand et al., 2019; Begar et al., 2025).

### Signaling in primary cilia

The close relationship between protein trafficking and signaling within the primary cilium has been further demonstrated by the proteomic profiling of the *Ift27* model cell line, where impaired protein trafficking was shown to result in an accumulation of ciliary GPCRs and associated signaling defects (Eguether et al., 2014; Liew et al., 2014). Subsequent studies have exploited the different temporal resolutions of proximity labeling methods to investigate dynamics in the primary cilium proteomes during active signaling (Liu et al., 2024; May et al., 2021). This enabled the identification of new components in the Sonic hedgehog signaling pathway, providing mechanistic insights. TurboID was used to identify the protein Numb as a positive regulator of hedgehog signaling (Liu et al., 2024). Numb localized at the cilia pocket and was found to be involved in the endocytosis of the hedgehog receptor Patched. We have used APEX2 to identify a negative regulator of hedgehog signaling, PALD1, which translocates into primary cilia after activation of hedgehog signaling and dampens the pathway in a cell type-specific manner (May et al., 2021). Moreover, our study captured known relocalizations of hedgehog components during active signaling on a proteomic level. Surprisingly, it also revealed the rapid removal of a regulatory subunit of protein kinase A (PKA) in response to hedgehog signaling, which sparked additional mechanistic studies on PKA signaling in cilia (May et al., 2021; Happ et al., 2022; Hoppe et al., 2024).

### Protein trafficking in cilia

As the signaling capacity of primary cilia is tightly linked to protein biogenesis, additional studies have exploited proximity labeling to investigate cilia protein trafficking mechanisms. To gain more insights into how ubiquitin regulates protein trafficking and protein quality control in cilia, one of our studies aimed to characterize the ciliary ubiquitinome (Aslanyan et al., 2023). Here, we targeted a BioID2 ubiquitin-binding domain (UBD) fusion protein to cilia and identified many components of the endosomal sorting complexes required for transport (ESCRT) complexes as well as proteins involved in clathrin-dependent endocytosis. These results confirmed the observation that activated GPCRs are removed from primary cilia via clathrin-mediated endocytosis (Pal et al., 2016) and indicate important roles of ubiquitin and the ESCRT machinery in the regulation of membrane processes in mIMCD3 cilia. In parallel, we used ubiquitin affinity proteomics to identify ubiquitin interactors in RPE1 cells, which suggested a ubiquitin-dependent turnover of caveolin-1 (CAV1) at the ciliary pocket that impacts cilia length regulation (Aslanyan et al., 2023). In a study aimed at gaining a deeper understanding of the loss of function of the BBSome, proteomic profiling of *Bbs3* (also known as *Arl6*) mutant cilia revealed an accumulation of ubiquitin and ubiquitin adaptors (Shinde et al., 2023). Dissecting the mechanism of ubiquitin-dependent protein removal by the BBSome revealed that the ubiquitin adaptor TOM1L2 recognizes K63-linked ubiquitin chains on cargos and bridged their interaction to the BBSome for IFT-dependent removal (Shinde et al., 2023, 2020; Desai et al., 2020). Other specific investigations focusing on disease mechanisms were carried out to study the function of DLG1 in trafficking polycystin-2 to primary cilia by BioID2 (Rezi et al., 2024), which suggested retromer-mediated transport from the Golgi.

### Extracellular vesicles in ciliary biology

Extracellular vesicles (EVs) have emerged as key players in cell and cilia biology, serving as vehicles for communication and cargo transport (Ngo et al., 2025). Initial comparative analyses of ciliary membranes and ectosomes from *C. rheinhardtii* emphasized the unique lipid and protein composition of EVs shed from cilia and suggested specialized functions in cellular signaling and homeostasis (Wood et al., 2013; Long et al., 2016). Later studies in mammalian systems have confirmed that EV release plays an important role in regulating the ciliary proteome, and that ciliary membrane remodeling and excision are actin dependent (Nager et al., 2017; Phua et al., 2017; Prasai et al., 2025). Earlier work in *C. elegans* was based on biochemical isolation and proteomic profiling of EVs and revealed their dynamic cargo composition and potential signaling roles (Nikonorova et al., 2022). More recent TurboID-based proximity proteomics allowed the specific analysis of polycystin-containing, cilia-derived EVs, which suggested a complex level of regulation in EV biogenesis and function (Nikonorova et al., 2025). Together, these findings underscore the importance of EVs in modulating ciliary function and signaling across diverse organisms.

Although the examples presented highlight the great potential of different technologies, it also becomes clear that the methods vary and have been used in a limited number of biological systems. This might be due to the limitations of the respective technologies – BioID-based approaches have a limited temporal resolution and APEX requires hydrogen peroxide as a substrate, which is not only toxic but also activates non-ciliary peroxidases that create significant background (Sroka et al., 2025 preprint). This background increases with the amounts of endogenous peroxidases, making downstream analysis of specific cell types very challenging. Our recent work has overcome this problem by locally activating APEX2 directly in primary cilia through a hydrogen peroxide-generating enzyme – D-amino acid oxidase (DAAO) (Pollegioni and Molla, 2011) – in a new technique termed *in situ* APEX activation (iAPEX) (Sroka et al., 2025 preprint). In a first proof-of-principle, iAPEX allowed not only *in vivo* applications in the model *Xenopus laevis,* but also a systematic comparison of the cilia proteomes of two different cell lines, mIMCD3 and NIH/3T3. Whereas core cilia components involved in protein trafficking and signaling, such as IFTs, kinesins and dyneins were found in both cell types, interesting differences, for example in microtubule associated proteins, could be observed (Sroka et al., 2025 preprint). These differences might point towards a molecular basis for structural and functional differences in primary cilia of different cell types. Limited sensitivity might still account for some differences, however, dissimilarities in the protein compositions of primary cilia across cell types has long been known and has been proposed to be the basis for the broad spectra of symptoms in ciliopathies.

## Perspectives

If we imagine, as a thought experiment, that all the methodologies described above have successfully created a comprehensive picture of the protein composition of primary cilia, what would the subsequent steps be? To begin with, it is questionable how accurately such a comprehensive model would reflect a single, specific cilium. Much like the cells from which they originate, cilia exhibit considerable diversity, and it is very likely that they differ significantly in composition and function depending on cell type, developmental stage and tissue contexts. Precisely uncovering and characterizing this diversity, however, seems feasible with the methods outlined above. Approaches with high temporal resolution, such as APEX2, could be advantageous for studying signaling and ciliary dynamics in simple cellular models. *In vivo* labeling techniques such as TurboID or iAPEX could prove beneficial in more complex, living model organisms. Specificity could be further increased by using split-TurboID (Cho et al., 2020), fusing two inactive fragments of TurboID to different targeting motifs or bait proteins. Moreover, complementary applications of co-immunoprecipitations (co-IPs) using highly specific antibodies combined with ‘off-the-shelf’ proximity labeling such as ProtA-TurboID could enhance these analyses (Santos-Barriopedro et al., 2021, 2023). The dynamics of ciliary trafficking and signaling pathways connecting cilia to organelles such as mitochondria or nuclei could be explored through tandem proximity-labeling techniques combining two enzymes and substrates. One such appropriate methodology, termed TransitID, has been recently described (Qin et al., 2023). In this method, TurboID is first employed for biotinylation within a specific compartment, followed by protein labeling with alkyne through APEX and the subsequent addition of azide-fluorescein via click chemistry. This approach enables investigation into protein exchange between cilia and other organelles. Ultimately, the most comprehensive approach for understanding ciliary diversity would involve generating proteomic profiles of individual cilia and ideally linking these data spatially to their respective tissue contexts. Recent emerging technologies, such as deep visual proteomics (Mund et al., 2022) and spatial optoproteomics (Chen et al., 2023 preprint), which integrate machine learning algorithms to optimize image recognition and data integration, might enable the next steps to achieve this goal.

In conclusion, advances in primary cilia proteomics have not only expanded our understanding of ciliary composition and function but also continue to drive innovation in methodology. We believe that the technologies currently available will be key to generating increasingly precise insights into cilia diversity and dynamics, shedding new light on the roles of this vital organelle in health and disease.

## References

[JCS264197C1] Aloy, P., Bottcher, B., Ceulemans, H., Leutwein, C., Mellwig, C., Fischer, S., Gavin, A. C., Bork, P., Superti-Furga, G., Serrano, L. et al. (2004). Structure-based assembly of protein complexes in yeast. *Science* 303, 2026-2029. 10.1126/science.1092645 15044803

[JCS264197C2] Ammar, C., Schessner, J. P., Willems, S., Michaelis, A. C. and Mann, M. (2023). Accurate label-free quantification by directLFQ to compare unlimited numbers of proteomes. *Mol. Cell. Proteomics* 22, 100581. 10.1016/j.mcpro.2023.100581 37225017 PMC10315922

[JCS264197C3] Ansley, S. J., Badano, J. L., Blacque, O. E., Hill, J., Hoskins, B. E., Leitch, C. C., Kim, J. C., Ross, A. J., Eichers, E. R., Teslovich, T. M. et al. (2003). Basal body dysfunction is a likely cause of pleiotropic Bardet-Biedl syndrome. *Nature* 425, 628-633. 10.1038/nature02030 14520415

[JCS264197C139] Arts, H. H., Doherty, D., van Beersum, S. E., Parisi, M. A., Letteboer, S. J., Gorden, N. T., Peters, T. A., Märker, T., Voesenek, K., Kartono, A. et al. (2007). Mutations in the gene encoding the basal body protein RPGRIP1L, a nephrocystin-4 interactor, cause Joubert syndrome. *Nat Genet.* 39, 882-888. 10.1038/ng2069 17558407

[JCS264197C4] Aslanyan, M. G., Doornbos, C., Diwan, G. D., Anvarian, Z., Beyer, T., Junger, K., van Beersum, S. E. C., Russell, R. B., Ueffing, M., Ludwig, A. et al. (2023). A targeted multi- proteomics approach generates a blueprint of the ciliary ubiquitinome. *Front. Cell Dev. Biol.* 11, 1113656. 10.3389/fcell.2023.1113656 36776558 PMC9908615

[JCS264197C5] Avidor-Reiss, T., Maer, A. M., Koundakjian, E., Polyanovsky, A., Keil, T., Subramaniam, S. and Zuker, C. S. (2004). Decoding cilia function: defining specialized genes required for compartmentalized cilia biogenesis. *Cell* 117, 527-539. 10.1016/S0092-8674(04)00412-X 15137945

[JCS264197C6] Barabasi, A. L. and Oltvai, Z. N. (2004). Network biology: understanding the cell's functional organization. *Nat. Rev. Genet.* 5, 101-113. 10.1038/nrg1272 14735121

[JCS264197C7] Barabasi, A. L., Gulbahce, N. and Loscalzo, J. (2011). Network medicine: a network- based approach to human disease. *Nat. Rev. Genet.* 12, 56-68. 10.1038/nrg2918 21164525 PMC3140052

[JCS264197C8] Barr, M. M., DeModena, J., Braun, D., Nguyen, C. Q., Hall, D. H. and Sternberg, P. W. (2001). The Caenorhabditis elegans autosomal dominant polycystic kidney disease gene homologs lov-1 and pkd-2 act in the same pathway. *Curr. Biol.* 11, 1341-1346. 10.1016/S0960-9822(01)00423-7 11553327

[JCS264197C9] Barr, M. M. and Sternberg, P. W. (1999). A polycystic kidney-disease gene homologue required for male mating behaviour in C. elegans. *Nature* 401, 386-389. 10.1038/43913 10517638

[JCS264197C10] Begar, E., Seyrek, E. and Firat-Karalar, E. N. (2025). Navigating centriolar satellites: the role of PCM1 in cellular and organismal processes. *FEBS J.* 292, 688-708. 10.1111/febs.17194 38825736 PMC11839937

[JCS264197C11] Beyer, T., Bolz, S., Junger, K., Horn, N., Moniruzzaman, M., Wissinger, Y., Ueffing, M. and Boldt, K. (2018). CRISPR/Cas9-mediated genomic editing of Cluap1/IFT38 reveals a new role in actin arrangement. *Mol. Cell. Proteomics* 17, 1285-1294. 10.1074/mcp.RA117.000487 29615496 PMC6030719

[JCS264197C12] Beyer, T., Martins, T., Srikaran, J. J., Seda, M., Peskett, E., Klose, F., Junger, K., Beales, P. L., Ueffing, M., Boldt, K. et al. (2024). Affinity purification of intraflagellar Transport (IFT) proteins in mice using endogenous streptavidin/FLAG Tags. *Methods Mol. Biol.* 2725, 199-212. 10.1007/978-1-0716-3507-0_12 37856026

[JCS264197C13] Blackburn, K., Bustamante-Marin, X., Yin, W., Goshe, M. B. and Ostrowski, L. E. (2017). Quantitative proteomic analysis of human airway cilia identifies previously uncharacterized proteins of high abundance. *J. Proteome Res.* 16, 1579-1592. 10.1021/acs.jproteome.6b00972 28282151 PMC5733142

[JCS264197C14] Boesger, J., Wagner, V., Weisheit, W. and Mittag, M. (2009). Analysis of flagellar phosphoproteins from Chlamydomonas reinhardtii. *Eukaryot. Cell* 8, 922-932. 10.1128/EC.00067-09 19429781 PMC2708455

[JCS264197C15] Boldt, K., Mans, D. A., Won, J., van Reeuwijk, J., Vogt, A., Kinkl, N., Letteboer, S. J., Hicks, W. L., Hurd, R. E., Naggert, J. K. et al. (2011). Disruption of intraflagellar protein transport in photoreceptor cilia causes Leber congenital amaurosis in humans and mice. *J. Clin. Investig.* 121, 2169-2180. 10.1172/JCI45627 21606596 PMC3104757

[JCS264197C16] Boldt, K., van Reeuwijk, J., Gloeckner, C. J., Ueffing, M. and Roepman, R. (2009). Tandem affinity purification of ciliopathy-associated protein complexes. *Methods Cell Biol.* 91, 143-160. 10.1016/S0091-679X(08)91009-8 20409786

[JCS264197C17] Boldt, K., van Reeuwijk, J., Lu, Q., Koutroumpas, K., Nguyen, T. M., Texier, Y., van Beersum, S. E., Horn, N., Willer, J. R., Mans, D. A. et al. (2016). An organelle-specific protein landscape identifies novel diseases and molecular mechanisms. *Nat. Commun.* 7, 11491. 10.1038/ncomms11491 27173435 PMC4869170

[JCS264197C18] Bork, P., Jensen, L. J., von Mering, C., Ramani, A. K., Lee, I. and Marcotte, E. M. (2004). Protein interaction networks from yeast to human. *Curr. Opin. Struct. Biol.* 14, 292-299. 10.1016/j.sbi.2004.05.003 15193308

[JCS264197C19] Branon, T. C., Bosch, J. A., Sanchez, A. D., Udeshi, N. D., Svinkina, T., Carr, S. A., Feldman, J. L., Perrimon, N. and Ting, A. Y. (2018). Efficient proximity labeling in living cells and organisms with TurboID. *Nat. Biotechnol.* 36, 880-887. 10.1038/nbt.4201 30125270 PMC6126969

[JCS264197C20] Breslow, D. K., Hoogendoorn, S., Kopp, A. R., Morgens, D. W., Vu, B. K., Kennedy, M. C., Han, K., Li, A., Hess, G. T., Bassik, M. C. et al. (2018). A CRISPR-based screen for Hedgehog signaling provides insights into ciliary function and ciliopathies. *Nat. Genet.* 50, 460-471. 10.1038/s41588-018-0054-7 29459677 PMC5862771

[JCS264197C21] Broadhead, R., Dawe, H. R., Farr, H., Griffiths, S., Hart, S. R., Portman, N., Shaw, M. K., Ginger, M. L., Gaskell, S. J., McKean, P. G. et al. (2006). Flagellar motility is required for the viability of the bloodstream trypanosome. *Nature* 440, 224-227. 10.1038/nature04541 16525475

[JCS264197C22] Chen, Y.-D., Chang, C.-W., Cheung, C. H. Y., Chang, H.-J., Sie, Y.-D., Chung, C.-W., Huang, C.-K., Huang, C.-C., Chong, W. M., Liu, Y.-P. et al. (2023). Microscopy-guided subcellular proteomic discovery by high-speed ultra-content photo-biotinylation. *bioRxiv*, 2023.12.27.573388. 10.1101/2023.12.27.573388

[JCS264197C23] Chih, B., Liu, P., Chinn, Y., Chalouni, C., Komuves, L. G., Hass, P. E., Sandoval, W. and Peterson, A. S. (2011). A ciliopathy complex at the transition zone protects the cilia as a privileged membrane domain. *Nat. Cell Biol.* 14, 61-72. 10.1038/ncb2410 22179047

[JCS264197C24] Cho, K. F., Branon, T. C., Rajeev, S., Svinkina, T., Udeshi, N. D., Thoudam, T., Kwak, C., Rhee, H.-W., Lee, I.-K., Carr, S. A. et al. (2020). Split-TurboID enables contact-dependent proximity labeling in cells. *Proc. Natl Acad. Sci. USA* 117, 12143-12154. 10.1073/pnas.1919528117 32424107 PMC7275672

[JCS264197C25] Cole, D. G., Diener, D. R., Himelblau, A. L., Beech, P. L., Fuster, J. C. and Rosenbaum, J. L. (1998). Chlamydomonas kinesin-II-dependent intraflagellar transport (IFT): IFT particles contain proteins required for ciliary assembly in Caenorhabditis elegans sensory neurons. *J. Cell Biol.* 141, 993-1008. 10.1083/jcb.141.4.993 9585417 PMC2132775

[JCS264197C26] Dai, D., Ichikawa, M., Peri, K., Rebinsky, R. and Huy Bui, K. (2020). Identification and mapping of central pair proteins by proteomic analysis. *Biophys. Physicobiol.* 17, 71-85. 10.2142/biophysico.BSJ-2019048 33178545 PMC7596323

[JCS264197C28] den Hollander, A. I., Koenekoop, R. K., Mohamed, M. D., Arts, H. H., Boldt, K., Towns, K. V., Sedmak, T., Beer, M., Nagel-Wolfrum, K., McKibbin, M. et al. (2007). Mutations in LCA5, encoding the ciliary protein lebercilin, cause Leber congenital amaurosis. *Nat. Genet.* 39, 889-895. 10.1038/ng2066 17546029

[JCS264197C29] Desai, P. B., Stuck, M. W., Lv, B. and Pazour, G. J. (2020). Ubiquitin links smoothened to intraflagellar transport to regulate Hedgehog signaling. *J. Cell Biol.* 219, e201912104. 10.1083/jcb.201912104 32435793 PMC7337509

[JCS264197C30] Diener, D. R., Lupetti, P. and Rosenbaum, J. L. (2015). Proteomic analysis of isolated ciliary transition zones reveals the presence of ESCRT proteins. *Curr. Biol.* 25, 379-384. 10.1016/j.cub.2014.11.066 25578910 PMC4318714

[JCS264197C140] Eblimit, A., Nguyen, T. M., Chen, Y., Esteve-Rudd, J., Zhong, H., Letteboer, S., Van Reeuwijk, J., Simons, D. L., Ding, Q., Wu, K.M. et al. (2015). Spata7 is a retinal ciliopathy gene critical for correct RPGRIP1 localization and protein trafficking in the retina. *Hum. Mol. Genet.* 24, 1584-1601. 10.1093/hmg/ddu573 25398945 PMC4351378

[JCS264197C31] Eguether, T., San Agustin, J. T., Keady, B. T., Jonassen, J. A., Liang, Y., Francis, R., Tobita, K., Johnson, C. A., Abdelhamed, Z. A., Lo, C. W. et al. (2014). IFT27 links the BBSome to IFT for maintenance of the ciliary signaling compartment. *Dev. Cell* 31, 279-290. 10.1016/j.devcel.2014.09.011 25446516 PMC4254547

[JCS264197C32] Faber, S., Mercey, O., Junger, K., Garanto, A., May-Simera, H., Ueffing, M., Collin, R. W., Boldt, K., Guichard, P., Hamel, V. et al. (2023). Gene augmentation of LCA5-associated Leber congenital amaurosis ameliorates bulge region defects of the photoreceptor ciliary axoneme. *JCI Insight* 8, e169162. 10.1172/jci.insight.169162 37071472 PMC10322687

[JCS264197C33] Failler, M., Giro-Perafita, A., Owa, M., Srivastava, S., Yun, C., Kahler, D. J., Unutmaz, D., Esteva, F. J., Sanchez, I. and Dynlacht, B. D. (2021). Whole-genome screen identifies diverse pathways that negatively regulate ciliogenesis. *Mol. Biol. Cell* 32, 169-185. 10.1091/mbc.E20-02-0111 33206585 PMC8120696

[JCS264197C34] Firat-Karalar, E. N., Rauniyar, N., Yates, J. R., III and Stearns, T. (2014). Proximity interactions among centrosome components identify regulators of centriole duplication. *Curr. Biol.* 24, 664-670. 10.1016/j.cub.2014.01.067 24613305 PMC4004176

[JCS264197C35] Garcia-Gonzalo, F. R., Corbit, K. C., Sirerol-Piquer, M. S., Ramaswami, G., Otto, E. A., Noriega, T. R., Seol, A. D., Robinson, J. F., Bennett, C. L., Josifova, D. J. et al. (2011). A transition zone complex regulates mammalian ciliogenesis and ciliary membrane composition. *Nat. Genet.* 43, 776-784. 10.1038/ng.891 21725307 PMC3145011

[JCS264197C36] Gavin, A. C., Aloy, P., Grandi, P., Krause, R., Boesche, M., Marzioch, M., Rau, C., Jensen, L. J., Bastuck, S., Dumpelfeld, B. et al. (2006). Proteome survey reveals modularity of the yeast cell machinery. *Nature* 440, 631-636. 10.1038/nature04532 16429126

[JCS264197C37] Gheiratmand, L., Coyaud, E., Gupta, G. D., Laurent, E. M., Hasegan, M., Prosser, S. L., Gonçalves, J., Raught, B. and Pelletier, L. (2019). Spatial and proteomic profiling reveals centrosome-independent features of centriolar satellites. *EMBO J.* 38, e101109. 10.15252/embj.2018101109 31304627 PMC6627244

[JCS264197C38] Gherman, A., Davis, E. E. and Katsanis, N. (2006). The ciliary proteome database: an integrated community resource for the genetic and functional dissection of cilia. *Nat. Genet.* 38, 961-962. 10.1038/ng0906-961 16940995

[JCS264197C39] Gibson, T. J., Seiler, M. and Veitia, R. A. (2013). The transience of transient overexpression. *Nat. Methods* 10, 715-721. 10.1038/nmeth.2534 23900254

[JCS264197C40] Gingras, A. C., Aebersold, R. and Raught, B. (2005). Advances in protein complex analysis using mass spectrometry. *J. Physiol.* 563, 11-21. 10.1113/jphysiol.2004.080440 15611014 PMC1665575

[JCS264197C41] Gloeckner, C. J., Boldt, K., Schumacher, A., Roepman, R. and Ueffing, M. (2007). A novel tandem affinity purification strategy for the efficient isolation and characterisation of native protein complexes. *Proteomics* 7, 4228-4234. 10.1002/pmic.200700038 17979178

[JCS264197C42] Gupta, G. D., Coyaud, E., Goncalves, J., Mojarad, B. A., Liu, Y., Wu, Q., Gheiratmand, L., Comartin, D., Tkach, J. M., Cheung, S. W. et al. (2015). A dynamic protein interaction landscape of the human centrosome-cilium interface. *Cell* 163, 1484-1499. 10.1016/j.cell.2015.10.065 26638075 PMC5089374

[JCS264197C43] Guzman, U. H., Martinez-Val, A., Ye, Z., Damoc, E., Arrey, T. N., Pashkova, A., Renuse, S., Denisov, E., Petzoldt, J., Peterson, A. C. et al. (2024). Ultra-fast label-free quantification and comprehensive proteome coverage with narrow-window data-independent acquisition. *Nat. Biotechnol.* 42, 1855-1866. 10.1038/s41587-023-02099-7 38302753 PMC11631760

[JCS264197C44] Hansen, J. N., Sun, H., Kahnert, K., Westenius, E., Johannesson, A., Villegas, C., Le, T., Tzavlaki, K., Winsnes, C. et al. (2025). Intrinsic heterogeneity of primary cilia revealed through spatial proteomics. *Cell*, S0092-8674(25)01029-3. 10.1016/j.cell.2025.08.039 41005307

[JCS264197C45] Happ, J. T., Arveseth, C. D., Bruystens, J., Bertinetti, D., Nelson, I. B., Olivieri, C., Zhang, J., Hedeen, D. S., Zhu, J. F., Capener, J. L. et al. (2022). A PKA inhibitor motif within SMOOTHENED controls Hedgehog signal transduction. *Nat. Struct. Mol. Biol.* 29, 990-999. 10.1038/s41594-022-00838-z 36202993 PMC9696579

[JCS264197C46] Hartwell, L. H., Hopfield, J. J., Leibler, S. and Murray, A. W. (1999). From molecular to modular cell biology. *Nature* 402, C47-C52. 10.1038/35011540 10591225

[JCS264197C47] Hildebrandt, F., Benzing, T. and Katsanis, N. (2011). Ciliopathies. *N. Engl. J. Med.* 364, 1533-1543. 10.1056/NEJMra1010172 21506742 PMC3640822

[JCS264197C48] Ho, K. H., Candat, A., Scarpetta, V., Faucourt, M., Weill, S., Salio, C., D'Este, E., Meschkat, M., Wurm, C. A., Kneussel, M. et al. (2023). Choroid plexuses carry nodal-like cilia that undergo axoneme regression from early adult stage. *Dev. Cell* 58, 2641-2651.e6. 10.1016/j.devcel.2023.10.003 37890489

[JCS264197C49] Hoppe, N., Harrison, S., Hwang, S. H., Chen, Z., Karelina, M., Deshpande, I., Suomivuori, C. M., Palicharla, V. R., Berry, S. P., Tschaikner, P. et al. (2024). GPR161 structure uncovers the redundant role of sterol-regulated ciliary cAMP signaling in the Hedgehog pathway. *Nat. Struct. Mol. Biol.* 31, 667-677. 10.1038/s41594-024-01223-8 38326651 PMC11221913

[JCS264197C50] Huang, B., Masyuk, T. and LaRusso, N. (2009). Isolation of primary cilia for morphological analysis. In *Primary Cilia*, Vol. 94 (ed. R. D. Sloboda), pp. 103-115: Academic Press. 10.1016/S0091-679X(08)94005-X 20362087

[JCS264197C51] Huttlin, E. L., Ting, L., Bruckner, R. J., Gebreab, F., Gygi, M. P., Szpyt, J., Tam, S., Zarraga, G., Colby, G., Baltier, K. et al. (2015). The BioPlex network: a systematic exploration of the human interactome. *Cell* 162, 425-440. 10.1016/j.cell.2015.06.043 26186194 PMC4617211

[JCS264197C52] Iyer, S. S., Chen, F., Ogunmolu, F. E., Moradi, S., Volkov, V. A., van Grinsven, E. J., van Hoorn, C., Wu, J., Andrea, N., Hua, S. et al. (2025). Centriolar cap proteins CP110 and CPAP control slow elongation of microtubule plus ends. *J. Cell Biol.* 224, e202406061. 10.1083/jcb.202406061 39847124 PMC11756378

[JCS264197C53] Ishikawa, H. and Marshall, W. F. (2013). Chapter fifteen - isolation of mammalian primary cilia. In *Methods in Enzymology*, Vol. 525 (ed. W. F. Marshall), pp. 311-325: Academic Press. 10.1016/B978-0-12-397944-5.00015-8 23522476

[JCS264197C54] Ishikawa, H., Thompson, J., Yates, J. R., III and Marshall, W. F. (2012). Proteomic analysis of mammalian primary cilia. *Curr. Biol.* 22, 414-419. 10.1016/j.cub.2012.01.031 22326026 PMC3298568

[JCS264197C55] Jiang, Y., Rex, D. A. B., Schuster, D., Neely, B. A., Rosano, G. L., Volkmar, N., Momenzadeh, A., Peters-Clarke, T. M., Egbert, S. B., Kreimer, S. et al. (2024). Comprehensive overview of bottom-up proteomics using mass spectrometry. *ACS Meas. Sci. Au.* 4, 338-417. 10.1021/acsmeasuresciau.3c00068 39193565 PMC11348894

[JCS264197C56] Kiesel, P., Alvarez Viar, G., Tsoy, N., Maraspini, R., Gorilak, P., Varga, V., Honigmann, A. and Pigino, G. (2020). The molecular structure of mammalian primary cilia revealed by cryo- electron tomography. *Nat. Struct. Mol. Biol.* 27, 1115-1124. 10.1038/s41594-020-0507-4 32989303 PMC7610599

[JCS264197C57] Kim, S. and Dynlacht, B. D. (2013). Assembling a primary cilium. *Curr. Opin. Cell Biol.* 25, 506-511. 10.1016/j.ceb.2013.04.011 23747070 PMC3729615

[JCS264197C58] Kim, J. H., Ki, S. M., Joung, J. G., Scott, E., Heynen-Genel, S., Aza-Blanc, P., Kwon, C. H., Kim, J., Gleeson, J. G. and Lee, J. E. (2016). Genome-wide screen identifies novel machineries required for both ciliogenesis and cell cycle arrest upon serum starvation. *Biochim. Biophys. Acta* 1863, 1307-1318. 10.1016/j.bbamcr.2016.03.021 27033521 PMC4886714

[JCS264197C59] Klimmeck, D., Mayer, U., Ungerer, N., Warnken, U., Schnolzer, M., Frings, S. and Mohrlen, F. (2008). Calcium-signaling networks in olfactory receptor neurons. *Neuroscience* 151, 901-912. 10.1016/j.neuroscience.2007.11.023 18155848

[JCS264197C60] Kohli, P., Hohne, M., Jungst, C., Bertsch, S., Ebert, L. K., Schauss, A. C., Benzing, T., Rinschen, M. M. and Schermer, B. (2017). The ciliary membrane-associated proteome reveals actin-binding proteins as key components of cilia. *EMBO Rep.* 18, 1521-1535. 10.15252/embr.201643846 28710093 PMC5579364

[JCS264197C61] Kuhlmann, K., Tschapek, A., Wiese, H., Eisenacher, M., Meyer, H. E., Hatt, H. H., Oeljeklaus, S. and Warscheid, B. (2014). The membrane proteome of sensory cilia to the depth of olfactory receptors. *Mol. Cell. Proteomics* 13, 1828-1843. 10.1074/mcp.M113.035378 24748648 PMC4083118

[JCS264197C62] Lai, C. K., Gupta, N., Wen, X., Rangell, L., Chih, B., Peterson, A. S., Bazan, J. F., Li, L. and Scales, S. J. (2011). Functional characterization of putative cilia genes by high-content analysis. *Mol. Biol. Cell* 22, 1104-1119. 10.1091/mbc.e10-07-0596 21289087 PMC3069013

[JCS264197C63] Lam, S. S., Martell, J. D., Kamer, K. J., Deerinck, T. J., Ellisman, M. H., Mootha, V. K. and Ting, A. Y. (2015). Directed evolution of APEX2 for electron microscopy and proximity labeling. *Nat. Methods* 12, 51-54. 10.1038/nmeth.3179 25419960 PMC4296904

[JCS264197C64] Langousis, G., Cavadini, S., Boegholm, N., Lorentzen, E., Kempf, G. and Matthias, P. (2022). Structure of the ciliogenesis-associated CPLANE complex. *Sci. Adv.* 8, eabn0832. 10.1126/sciadv.abn0832 35427153 PMC9012472

[JCS264197C65] Latour, B. L., Van De Weghe, J. C., Rusterholz, T. D., Letteboer, S. J., Gomez, A., Shaheen, R., Gesemann, M., Karamzade, A., Asadollahi, M., Barroso-Gil, M. et al. (2020). Dysfunction of the ciliary ARMC9/TOGARAM1 protein module causes Joubert syndrome. *J. Clin. Investig.* 130, 4423-4439. 10.1172/JCI131656 32453716 PMC7410078

[JCS264197C66] Leung, M. R., Sun, C., Zeng, J., Anderson, J. R., Niu, Q., Huang, W., Noteborn, W. E. M., Brown, A., Zeev-Ben-Mordehai, T. and Zhang, R. (2025). Structural diversity of axonemes across mammalian motile cilia. *Nature* 637, 1170-1177. 10.1038/s41586-024-08337-5 39743588 PMC11779644

[JCS264197C67] Li, J. B., Gerdes, J. M., Haycraft, C. J., Fan, Y., Teslovich, T. M., May-Simera, H., Li, H., Blacque, O. E., Li, L., Leitch, C. C. et al. (2004). Comparative genomics identifies a flagellar and basal body proteome that includes the BBS5 human disease gene. *Cell* 117, 541-552. 10.1016/S0092-8674(04)00450-7 15137946

[JCS264197C68] Li, J., Cai, Z., Bomgarden, R. D., Pike, I., Kuhn, K., Rogers, J. C., Roberts, T. M., Gygi, S. P. and Paulo, J. A. (2021). TMTpro-18plex: The Expanded and Complete Set of TMTpro Reagents for Sample Multiplexing. *J. Proteome Res.* 20, 2964-2972. 10.1021/acs.jproteome.1c00168 33900084 PMC8210943

[JCS264197C69] Liew, G. M., Ye, F., Nager, A. R., Murphy, J. P., Lee, J. S., Aguiar, M., Breslow, D. K., Gygi, S. P. and Nachury, M. V. (2014). The intraflagellar transport protein IFT27 promotes BBSome exit from cilia through the GTPase ARL6/BBS3. *Dev. Cell* 31, 265-278. 10.1016/j.devcel.2014.09.004 25443296 PMC4255629

[JCS264197C70] Liu, Q., Tan, G., Levenkova, N., Li, T., Pugh, E. N., Jr, Rux, J. J., Speicher, D. W. and Pierce, E. A. (2007). The proteome of the mouse photoreceptor sensory cilium complex. *Mol. Cell. Proteomics* 6, 1299-1317. 10.1074/mcp.M700054-MCP200 17494944 PMC2128741

[JCS264197C71] Liu, X., Yam, P. T., Schlienger, S., Cai, E., Zhang, J., Chen, W. J., Torres Gutierrez, O., Jimenez Amilburu, V., Ramamurthy, V., Ting, A. Y. et al. (2024). Numb positively regulates Hedgehog signaling at the ciliary pocket. *Nat. Commun.* 15, 3365. 10.1038/s41467-024-47244-1 38664376 PMC11045789

[JCS264197C72] Loktev, A. V., Zhang, Q., Beck, J. S., Searby, C. C., Scheetz, T. E., Bazan, J. F., Slusarski, D. C., Sheffield, V. C., Jackson, P. K. and Nachury, M. V. (2008). A BBSome subunit links ciliogenesis, microtubule stability, and acetylation. *Dev. Cell* 15, 854-865. 10.1016/j.devcel.2008.11.001 19081074

[JCS264197C73] Long, H., Zhang, F., Xu, N., Liu, G., Diener, D. R., Rosenbaum, J. L. and Huang, K. (2016). Comparative analysis of ciliary membranes and ectosomes. *Curr. Biol.* 26, 3327-3335. 10.1016/j.cub.2016.09.055 27866888 PMC5173405

[JCS264197C74] May, E. A., Kalocsay, M., D'Auriac, I. G., Schuster, P. S., Gygi, S. P., Nachury, M. V. and Mick, D. U. (2021). Time-resolved proteomics profiling of the ciliary Hedgehog response. *J. Cell Biol.* 220, e202007207. 10.1083/jcb.202007207 33856408 PMC8054476

[JCS264197C75] Mayer, U., Ungerer, N., Klimmeck, D., Warnken, U., Schnolzer, M., Frings, S. and Mohrlen, F. (2008). Proteomic analysis of a membrane preparation from rat olfactory sensory cilia. *Chem. Senses* 33, 145-162. 10.1093/chemse/bjm073 18032372

[JCS264197C76] Mayer, U., Kuller, A., Daiber, P. C., Neudorf, I., Warnken, U., Schnolzer, M., Frings, S. and Mohrlen, F. (2009). The proteome of rat olfactory sensory cilia. *Proteomics* 9, 322-334. 10.1002/pmic.200800149 19086097

[JCS264197C77] McIntosh, J. R., Grishchuk, E. L., Morphew, M. K., Efremov, A. K., Zhudenkov, K., Volkov, V. A., Cheeseman, I. M., Desai, A., Mastronarde, D. N. and Ataullakhanov, F. I. (2008). Fibrils connect microtubule tips with kinetochores: a mechanism to couple tubulin dynamics to chromosome motion. *Cell* 135, 322-333. 10.1016/j.cell.2008.08.038 18957206 PMC2746696

[JCS264197C78] Mick, D. U., Rodrigues, R. B., Leib, R. D., Adams, C. M., Chien, A. S., Gygi, S. P. and Nachury, M. V. (2015). Proteomics of primary cilia by proximity labeling. *Dev. Cell* 35, 497-512. 10.1016/j.devcel.2015.10.015 26585297 PMC4662609

[JCS264197C79] Mill, P., Christensen, S. T. and Pedersen, L. B. (2023). Primary cilia as dynamic and diverse signalling hubs in development and disease. *Nat. Rev. Genet.* 24, 421-441. 10.1038/s41576-023-00587-9 37072495 PMC7615029

[JCS264197C80] Mitchell, K. A. P. (2013). Isolation of primary cilia by shear force. *Curr. Protoc. Cell Biol.* 2013, Chapter 3:3.42.1-3.42.9. 10.1002/0471143030.cb0342s59 23728745

[JCS264197C81] Mitchell, K. A. P., Szabo, G. and Otero, A. D. S. (2009). Chapter 4 - Methods for the isolation of sensory and primary cilia: an overview. In *Methods in Cell Biology*, Vol. 94 (ed. R. D. Sloboda), pp. 87-101: Academic Press. 10.1016/S0091-679X(08)94004-8 20362086

[JCS264197C82] Moran, A. L., Louzao-Martinez, L., Norris, D. P., Peters, D. J. M. and Blacque, O. E. (2024). Transport and barrier mechanisms that regulate ciliary compartmentalization and ciliopathies. *Nat. Rev. Nephrol.* 20, 83-100. 10.1038/s41581-023-00773-2 37872350

[JCS264197C83] Moyer, J. H., Lee-Tischler, M. J., Kwon, H. Y., Schrick, J. J., Avner, E. D., Sweeney, W. E., Godfrey, V. L., Cacheiro, N. L., Wilkinson, J. E. and Woychik, R. P. (1994). Candidate gene associated with a mutation causing recessive polycystic kidney disease in mice. *Science* 264, 1329-1333. 10.1126/science.8191288 8191288

[JCS264197C84] Mukhopadhyay, S., Wen, X., Chih, B., Nelson, C. D., Lane, W. S., Scales, S. J. and Jackson, P. K. (2010). TULP3 bridges the IFT-A complex and membrane phosphoinositides to promote trafficking of G protein-coupled receptors into primary cilia. *Genes Dev.* 24, 2180-2193. 10.1101/gad.1966210 20889716 PMC2947770

[JCS264197C85] Mund, A., Coscia, F., Kriston, A., Hollandi, R., Kovács, F., Brunner, A. D., Migh, E., Schweizer, L., Santos, A., Bzorek, M. et al. (2022). Deep Visual Proteomics defines single-cell identity and heterogeneity. *Nat. Biotechnol.* 40, 1231-1240. 10.1038/s41587-022-01302-5 35590073 PMC9371970

[JCS264197C86] Nachury, M. V. and Mick, D. U. (2019). Establishing and regulating the composition of cilia for signal transduction. *Nat. Rev. Mol. Cell Biol.* 20, 389-405. 10.1038/s41580-019-0116-4 30948801 PMC6738346

[JCS264197C87] Nachury, M. V., Loktev, A. V., Zhang, Q., Westlake, C. J., Peranen, J., Merdes, A., Slusarski, D. C., Scheller, R. H., Bazan, J. F., Sheffield, V. C. et al. (2007). A core complex of BBS proteins cooperates with the GTPase Rab8 to promote ciliary membrane biogenesis. *Cell* 129, 1201-1213. 10.1016/j.cell.2007.03.053 17574030

[JCS264197C88] Nager, A. R., Goldstein, J. S., Herranz-Pérez, V., Portran, D., Ye, F., Garcia-Verdugo, J. M. and Nachury, M. V. (2017). An actin network dispatches ciliary GPCRs into extracellular vesicles to modulate signaling. *Cell* 168, 252-263.e14. 10.1016/j.cell.2016.11.036 28017328 PMC5235987

[JCS264197C89] Narita, K., Kozuka-Hata, H., Nonami, Y., Ao-Kondo, H., Suzuki, T., Nakamura, H., Yamakawa, K., Oyama, M., Inoue, T. and Takeda, S. (2012). Proteomic analysis of multiple primary cilia reveals a novel mode of ciliary development in mammals. *Biol. Open* 1, 815-825. 10.1242/bio.20121081 23213475 PMC3507226

[JCS264197C90] Ngo, J. M., Williams, J. K., Zhang, C., Saleh, A. H., Liu, X. M., Ma, L. and Schekman, R. (2025). Extracellular vesicles and cellular homeostasis. *Annu. Rev. Biochem.* 94, 587-609. 10.1146/annurev-biochem-100924-012717 40101210

[JCS264197C91] Nikonorova, I. A., Wang, J., Cope, A. L., Tilton, P. E., Power, K. M., Walsh, J. D., Akella, J. S., Krauchunas, A. R., Shah, P. and Barr, M. M. (2022). Isolation, profiling, and tracking of extracellular vesicle cargo in Caenorhabditis elegans. *Curr. Biol.* 32, 1924-1936.e6. 10.1016/j.cub.2022.03.005 35334227 PMC9491618

[JCS264197C92] Nikonorova, I. A., des Ranleau, E., Jacobs, K. C., Saul, J., Walsh, J. D., Wang, J. and Barr, M. M. (2025). Polycystins recruit cargo to distinct ciliary extracellular vesicle subtypes in C. elegans. *Nat. Commun.* 16, 2899. 10.1038/s41467-025-57512-3 40180912 PMC11968823

[JCS264197C93] Ostrowski, L. E., Blackburn, K., Radde, K. M., Moyer, M. B., Schlatzer, D. M., Moseley, A. and Boucher, R. C. (2002). A proteomic analysis of human cilia: identification of novel components. *Mol. Cell. Proteomics* 1, 451-465. 10.1074/mcp.M200037-MCP200 12169685

[JCS264197C94] Otto, E. A., Schermer, B., Obara, T., O'Toole, J. F., Hiller, K. S., Mueller, A. M., Ruf, R. G., Hoefele, J., Beekmann, F., Landau, D. et al. (2003). Mutations in INVS encoding inversin cause nephronophthisis type 2, linking renal cystic disease to the function of primary cilia and left-right axis determination. *Nat. Genet.* 34, 413-420. 10.1038/ng1217 12872123 PMC3732175

[JCS264197C95] Otto, E. A., Loeys, B., Khanna, H., Hellemans, J., Sudbrak, R., Fan, S., Muerb, U., O'Toole, J. F., Helou, J., Attanasio, M. et al. (2005). Nephrocystin-5, a ciliary IQ domain protein, is mutated in Senior-Loken syndrome and interacts with RPGR and calmodulin. *Nat. Genet.* 37, 282-288. 10.1038/ng1520 15723066

[JCS264197C96] Oud, M. M., Lamers, I. J. and Arts, H. H. (2017). Ciliopathies: genetics in pediatric medicine. *J. Pediatr. Genet.* 6, 18-29. 10.1055/s-0036-1593841 28180024 PMC5289266

[JCS264197C97] Pal, K., Hwang, S. H., Somatilaka, B., Badgandi, H., Jackson, P. K., DeFea, K. and Mukhopadhyay, S. (2016). Smoothened determines β-arrestin-mediated removal of the G protein-coupled receptor Gpr161 from the primary cilium. *J. Cell Biol.* 212, 861-875. 10.1083/jcb.201506132 27002170 PMC4810300

[JCS264197C98] Pan, J., Wang, Q. and Snell, W. J. (2005). Cilium-generated signaling and cilia-related disorders. *Lab. Invest.* 85, 452-463. 10.1038/labinvest.3700253 15723088

[JCS264197C99] Pazour, G. J., Dickert, B. L., Vucica, Y., Seeley, E. S., Rosenbaum, J. L., Witman, G. B. and Cole, D. G. (2000). Chlamydomonas IFT88 and its mouse homologue, polycystic kidney disease gene tg737, are required for assembly of cilia and flagella. *J. Cell Biol.* 151, 709-718. 10.1083/jcb.151.3.709 11062270 PMC2185580

[JCS264197C100] Pazour, G. J., Agrin, N., Leszyk, J. and Witman, G. B. (2005). Proteomic analysis of a eukaryotic cilium. *J. Cell Biol.* 170, 103-113. 10.1083/jcb.200504008 15998802 PMC2171396

[JCS264197C101] Phua, S. C., Chiba, S., Suzuki, M., Su, E., Roberson, E. C., Pusapati, G. V., Schurmans, S., Setou, M., Rohatgi, R., Reiter, J. F. et al. (2017). Dynamic remodeling of membrane composition drives cell cycle through primary cilia excision. *Cell* 168, 264-279.e15. 10.1016/j.cell.2016.12.032 28086093 PMC5660509

[JCS264197C102] Polino, A. J., Sviben, S., Melena, I., Piston, D. W. and Hughes, J. W. (2023). Scanning electron microscopy of human islet cilia. *Proc. Natl. Acad. Sci. USA* 120, e2302624120. 10.1073/pnas.2302624120 37205712 PMC10235940

[JCS264197C103] Pollegioni, L. and Molla, G. (2011). New biotech applications from evolved D-amino acid oxidases. *Trends Biotechnol.* 29, 276-283. 10.1016/j.tibtech.2011.01.010 21397351

[JCS264197C104] Prasai, A., Ivashchenko, O., Maskova, K., Bykova, S., Schmidt Cernohorska, M., Stepanek, O. and Huranova, M. (2025). BBSome-deficient cells activate intraciliary CDC42 to trigger actin-dependent ciliary ectocytosis. *EMBO Rep.* 26, 36-60. 10.1038/s44319-024-00326-z 39587330 PMC11724091

[JCS264197C105] Puig, O., Caspary, F., Rigaut, G., Rutz, B., Bouveret, E., Bragado-Nilsson, E., Wilm, M. and Seraphin, B. (2001). The tandem affinity purification (TAP) method: a general procedure of protein complex purification. *Methods* 24, 218-229. 10.1006/meth.2001.1183 11403571

[JCS264197C106] Pusapati, G. V., Kong, J. H., Patel, B. B., Krishnan, A., Sagner, A., Kinnebrew, M., Briscoe, J., Aravind, L. and Rohatgi, R. (2018). CRISPR Screens uncover genes that regulate target cell sensitivity to the morphogen sonic hedgehog. *Dev. Cell* 44, 113-129.e8. 10.1016/j.devcel.2017.12.003 29290584 PMC5792066

[JCS264197C107] Qin, W., Cheah, J. S., Xu, C., Messing, J., Freibaum, B. D., Boeynaems, S., Taylor, J. P., Udeshi, N. D., Carr, S. A. and Ting, A. Y. (2023). Dynamic mapping of proteome trafficking within and between living cells by TransitID. *Cell* 186, 3307-3324.e30. 10.1016/j.cell.2023.05.044 37385249 PMC10527209

[JCS264197C108] Quinlan, R. J., Tobin, J. L. and Beales, P. L. (2008). Modeling ciliopathies: primary cilia in development and disease. *Curr. Top. Dev. Biol.* 84, 249-310. 10.1016/S0070-2153(08)00605-4 19186246

[JCS264197C109] Rezi, C. K., Aslanyan, M. G., Diwan, G. D., Cheng, T., Chamlali, M., Junger, K., Anvarian, Z., Lorentzen, E., Pauly, K. B., Afshar-Bahadori, Y. et al. (2024). DLG1 functions upstream of SDCCAG3 and IFT20 to control ciliary targeting of polycystin-2. *EMBO Rep.* 25, 3040-3063. 10.1038/s44319-024-00170-1 38849673 PMC11239879

[JCS264197C110] Rhee, H. W., Zou, P., Udeshi, N. D., Martell, J. D., Mootha, V. K., Carr, S. A. and Ting, A. Y. (2013). Proteomic mapping of mitochondria in living cells via spatially restricted enzymatic tagging. *Science* 339, 1328-1331. 10.1126/science.1230593 23371551 PMC3916822

[JCS264197C111] Rigaut, G., Shevchenko, A., Rutz, B., Wilm, M., Mann, M. and Seraphin, B. (1999). A generic protein purification method for protein complex characterization and proteome exploration. *Nat. Biotechnol.* 17, 1030-1032. 10.1038/13732 10504710

[JCS264197C141] Roepman, R., Bernoud-Hubac, N., Schick, D. E., Maugeri, A., Berger, W., Ropers, H. H., Cremers, F. P. and Ferreira, P. A. (2000). The retinitis pigmentosa GTPase regulator (RPGR) interacts with novel transport-like proteins in the outer segments of rod photoreceptors. *Hum. Mol. Genet.* 9, 2095-2105. 10.1093/hmg/9.14.2095 10958648

[JCS264197C142] Roepman, R., Letteboer, S. J., Arts, H. H., van Beersum, S. E., Lu, X, Krieger, E., Ferreira, P. A. and Cremers, F. P. (2005). Interaction of nephrocystin-4 and RPGRIP1 is disrupted by nephronophthisis or Leber congenital amaurosis-associated mutations. *Proc. Natl. Acad. Sci. USA* 102, 18520-18525. 10.1073/pnas.0505774102 16339905 PMC1317916

[JCS264197C112] Roosing, S., Hofree, M., Kim, S., Scott, E., Copeland, B., Romani, M., Silhavy, J. L., Rosti, R. O., Schroth, J., Mazza, T. et al. (2015). Functional genome-wide siRNA screen identifies KIAA0586 as mutated in Joubert syndrome. *eLife* 4, e06602. 10.7554/eLife.06602 26026149 PMC4477441

[JCS264197C113] Roux, K. J., Kim, D. I., Raida, M. and Burke, B. (2012). A promiscuous biotin ligase fusion protein identifies proximal and interacting proteins in mammalian cells. *J. Cell Biol.* 196, 801-810. 10.1083/jcb.201112098 22412018 PMC3308701

[JCS264197C114] Rual, J. F., Venkatesan, K., Hao, T., Hirozane-Kishikawa, T., Dricot, A., Li, N., Berriz, G. F., Gibbons, F. D., Dreze, M., Ayivi-Guedehoussou, N. et al. (2005). Towards a proteome- scale map of the human protein-protein interaction network. *Nature* 437, 1173-1178. 10.1038/nature04209 16189514

[JCS264197C115] Sang, L., Miller, J. J., Corbit, K. C., Giles, R. H., Brauer, M. J., Otto, E. A., Baye, L. M., Wen, X., Scales, S. J., Kwong, M. et al. (2011). Mapping the NPHP-JBTS-MKS protein network reveals ciliopathy disease genes and pathways. *Cell* 145, 513-528. 10.1016/j.cell.2011.04.019 21565611 PMC3383065

[JCS264197C116] Santos-Barriopedro, I., van Mierlo, G. and Vermeulen, M. (2021). Off-the-shelf proximity biotinylation for interaction proteomics. *Nat. Commun.* 12, 5015. 10.1038/s41467-021-25338-4 34408139 PMC8373943

[JCS264197C117] Santos-Barriopedro, I., van Mierlo, G. and Vermeulen, M. (2023). Off-the-shelf proximity biotinylation using ProtA-TurboID. *Nat. Protoc.* 18, 36-57. 10.1038/s41596-022-00748-w 36224470

[JCS264197C118] Saunders, H. A. J., van den Berg, C. M., Hoogebeen, R. A., Schweizer, D., Stecker, K. E., Roepman, R., Howes, S. C. and Akhmanova, A. (2025). A network of interacting ciliary tip proteins with opposing activities imparts slow and processive microtubule growth. *Nat. Struct. Mol. Biol*. 32, 979-994. 10.1038/s41594-025-01483-y 39856351 PMC12170345

[JCS264197C119] Schellens, R. T. W., Slijkerman, R. W. N., Hetterschijt, L., Peters, T. A., Broekman, S., Clemént, A., Westerfield, M., Phillips, J. B., Boldt, K., Kremer, H. et al. (2022). Affinity purification of in vivo assembled whirlin-associated protein complexes from the zebrafish retina. *J. Proteomics* 266, 104666. 10.1016/j.jprot.2022.104666 35788411

[JCS264197C120] Shinde, S. R., Nager, A. R. and Nachury, M. V. (2020). Ubiquitin chains earmark GPCRs for BBSome-mediated removal from cilia. *J. Cell Biol.* 219, e202003020. 10.1083/jcb.202003020 33185668 PMC7716378

[JCS264197C121] Shinde, S. R., Mick, D. U., Aoki, E., Rodrigues, R. B., Gygi, S. P. and Nachury, M. V. (2023). The ancestral ESCRT protein TOM1L2 selects ubiquitinated cargoes for retrieval from cilia. *Dev. Cell* 58, 677-693.e9. 10.1016/j.devcel.2023.03.003 37019113 PMC10133032

[JCS264197C122] Sim, H. J., Yun, S., Kim, H. E., Kwon, K. Y., Kim, G. H., Yun, S., Kim, B. G., Myung, K., Park, T. J. and Kwon, T. (2020). Simple Method To Characterize the Ciliary Proteome of Multiciliated Cells. *J. Proteome Res.* 19, 391-400. 10.1021/acs.jproteome.9b00589 31689115

[JCS264197C123] Singla, V. and Reiter, J. F. (2006). The primary cilium as the cell's antenna: signaling at a sensory organelle. *Science* 313, 629-633. 10.1126/science.1124534 16888132

[JCS264197C124] Snider, J., Kotlyar, M., Saraon, P., Yao, Z., Jurisica, I. and Stagljar, I. (2015). Fundamentals of protein interaction network mapping. *Mol. Syst. Biol.* 11, 848. 10.15252/msb.20156351 26681426 PMC4704491

[JCS264197C125] Sroka, T. J., Sanwald, L. K., Prasai, A., Hoeren, J., von der Malsburg, K., Chaumet, V., Haberkant, P., Feistel, K. and Mick, D. U. (2025). iAPEX: Improved APEX-based proximity labeling for subcellular proteomics using an enzymatic reaction cascade. *bioRxiv*, 2025.01.10.632381. 10.1101/2025.01.10.632381

[JCS264197C126] Stephan, A. B., Shum, E. Y., Hirsh, S., Cygnar, K. D., Reisert, J. and Zhao, H. (2009). ANO2 is the cilial calcium-activated chloride channel that may mediate olfactory amplification. *Proc. Natl. Acad. Sci. USA* 106, 11776-11781. 10.1073/pnas.0903304106 19561302 PMC2702256

[JCS264197C127] Subota, I., Julkowska, D., Vincensini, L., Reeg, N., Buisson, J., Blisnick, T., Huet, D., Perrot, S., Santi-Rocca, J., Duchateau, M. et al. (2014). Proteomic analysis of intact flagella of procyclic Trypanosoma brucei cells identifies novel flagellar proteins with unique sub- localization and dynamics. *Mol. Cell. Proteomics* 13, 1769-1786. 10.1074/mcp.M113.033357 24741115 PMC4083114

[JCS264197C128] Szymanska, K., Wheway, G., Doherty, D., Schmidts, M., Mans, D., Nguyen, T. M. T., Boldt, K., Tödt, G., Abdelhamed, Z., Wunderlich, K. et al. (2015). A high-throughput genome- wide siRNA screen for ciliogenesis identifies new ciliary functional components and ciliopathy genes. *Cilia* 4, O12. 10.1186/2046-2530-4-S1-O12

[JCS264197C129] Toriyama, M., Lee, C., Taylor, S. P., Duran, I., Cohn, D. H., Bruel, A. L., Tabler, J. M., Drew, K., Kelly, M. R., Kim, S. et al. (2016). The ciliopathy-associated CPLANE proteins direct basal body recruitment of intraflagellar transport machinery. *Nat. Genet.* 48, 648-656. 10.1038/ng.3558 27158779 PMC4978421

[JCS264197C130] Turan, M. G., Orhan, M. E., Cevik, S. and Kaplan, O. I. (2023). CiliaMiner: an integrated database for ciliopathy genes and ciliopathies. *Database (Oxford)* 2023, baad047. 10.1093/database/baad047 37542408 PMC10403755

[JCS264197C131] Vazquez, N, Lee, C, Valenzuela, I, Phan, TP, Derderian, C, Chávez, M, Mooney, NA, Demeter, J, Aziz-Zanjani, MO, Cusco, I. et al. (2024). The human ciliopathy protein RSG1 links the CPLANE complex to transition zone architecture. *Nat. Commun.* 16, 5701. 10.1038/s41467-025-61005-8 PMC12219219 40593758

[JCS264197C132] Vidal, M., Cusick, M. E. and Barabasi, A. L. (2011). Interactome networks and human disease. *Cell* 144, 986-998. 10.1016/j.cell.2011.02.016 21414488 PMC3102045

[JCS264197C133] Walton, T., Gui, M., Velkova, S., Fassad, M. R., Hirst, R. A., Haarman, E., O'Callaghan, C., Bottier, M., Burgoyne, T., Mitchison, H. M. et al. (2023). Axonemal structures reveal mechanoregulatory and disease mechanisms. *Nature* 618, 625-633. 10.1038/s41586-023-06140-2 37258679 PMC10266980

[JCS264197C134] Wang, P. I. and Marcotte, E. M. (2010). It's the machine that matters: predicting gene function and phenotype from protein networks. *J. Proteomics* 73, 2277-2289. 10.1016/j.jprot.2010.07.005 20637909 PMC2953423

[JCS264197C135] Wang, L., Wen, X., Wang, Z., Lin, Z., Li, C., Zhou, H., Yu, H., Li, Y., Cheng, Y., Chen, Y. et al. (2022). Ciliary transition zone proteins coordinate ciliary protein composition and ectosome shedding. *Nat. Commun.* 13, 3997. 10.1038/s41467-022-31751-0 35810181 PMC9271036

[JCS264197C136] Wood, C. R., Huang, K., Diener, D. R. and Rosenbaum, J. L. (2013). The cilium secretes bioactive ectosomes. *Curr. Biol.* 23, 906-911. 10.1016/j.cub.2013.04.019 23623554 PMC3850760

[JCS264197C137] Zhang, T., Liu, X., Rossio, V., Dawson, S. L., Gygi, S. P. and Paulo, J. A. (2024). Enhancing proteome coverage by using strong anion-exchange in tandem with basic-pH reversed-phase chromatography for sample multiplexing-based proteomics. *J. Proteome Res.* 23, 2870-2881. 10.1021/acs.jproteome.3c00492 37962907 PMC11090996

[JCS264197C138] Zhao, L., Hou, Y., Picariello, T., Craige, B. and Witman, G. B. (2019). Proteome of the central apparatus of a ciliary axoneme. *J. Cell Biol.* 218, 2051-2070. 10.1083/jcb.201902017 31092556 PMC6548120

